# Effectivity of mesenchymal stem cells for bleomycin-induced pulmonary fibrosis: a systematic review and implication for clinical application

**DOI:** 10.1186/s13287-021-02551-y

**Published:** 2021-08-23

**Authors:** Yunyu Zhao, Zhipeng Yan, Ying Liu, Yue Zhang, Jie Shi, Jingtao Li, Fanpu Ji

**Affiliations:** 1 Department of Infectious Diseases, The Second Affiliated Hospital of Xi’an Jiaotong University, 157 Xi Wu Road, Xi’an, 710004 Shaanxi China; 2 Department of Liver Diseases, The Hospital Affiliated to Shaanxi University of Chinese Medicine, Xianyang, 712046 China; 3 The Second Affiliated Hospital of Xi’an Jiaotong University, Xi’an, 710004 China; 4 Department of Respiratory, The Hospital Affiliated to Shaanxi University of Chinese Medicine, Xianyang, China; 5 National and Local Joint Engineering Research Center of Biodiagnosis and Biotherapy, The Second Affiliated Hospital of Xi’an Jiaotong University, Xi’an, China; 6 Key Laboratory of Environment and Genes Related To Diseases, Xi’an Jiaotong University, Ministry of Education of China, Xi’an, China

**Keywords:** Pulmonary fibrosis, Mesenchymal stem cells, COVID-19, SARS-CoV-2, Bleomycin, Systematic review

## Abstract

Pulmonary fibrosis (PF) is a chronic, progressive, fibrotic interstitial disease of the lung with poor prognosis and without effective treatment currently. Data from previous coronavirus infections, such as the Severe Acute Respiratory Syndrome (SARS) and Middle East Respiratory Syndrome, as well as current clinical evidence from the Coronavirus disease 2019 (COVID-19), support that SARS-CoV-2 infection may lead to PF, seriously impacting patient prognosis and quality of life. Therefore, effective prevention and treatment of PF will improve patient prognosis and reduce the overall social and economic burdens. Stem cells, especially mesenchymal stem cells (MSCs) have many great advantages, including migration to damaged lung tissue and secretion of various paracrine factors, thereby regulating the permeability of endothelial and epithelial cells, reducing inflammatory response, promoting tissue repair and inhibiting bacterial growth. Clinical trials of MSCs for the treatment of acute lung injury, PF and severe and critically ill COVID-19 are ongoing. The purpose of this study is to systematically review preclinical studies, explored the effectiveness of MSCs in the treatment of bleomycin (BLM)-induced pulmonary fibrosis and analyze the potential mechanism, combined with clinical trials of current MSCs for idiopathic pulmonary fibrosis (IPF) and COVID-19, so as to provide support for clinical research and transformation of MSCs. Searching PubMed and Embase (− 2021.4) identified a total of 36 preclinical studies of MSCs as treatment of BLM-induced acute lung injury and PF in rodent models. Most of the studies showed the MSCs treatment to reduce BLM-induced lung tissue inflammatory response, inflammatory cell infiltration, inflammatory cytokine expression, extracellular matrix production and collagen deposition, and to improve Ashcroft score. The results of present studies indicate that MSCs may serve as a potential therapeutic modality for the treatment of PF, including viral-induced PF and IPF.

## Introduction

Pulmonary fibrosis (PF) is a chronic, progressive and irreversible condition in interstitial lung diseases (ILDs). Its pathological features are dysfunctional repair of normal lung tissue after damage, excessive proliferation of fibroblasts and large amounts of collagen deposition, which can collectively lead to decreased lung compliance, blocked gas exchange and, ultimately, respiratory failure and death [1]. The etiology of PF is complex and related to environment, drug, pathogenesis, genetic and other factors. Although the etiologies vary for different pathologic forms of PF, the current hypothesis of its general pathogenesis involves abnormal wound healing of alveolar epithelial cells in response to repeated injury stimulation [2]. Injured alveolar epithelial cells secrete a variety of immune mediators, including cytokines, growth factors, and chemokines, such as TGF-β, IL-1β, IL-2, IL-6, and matrix metalloproteinases (MMPs), that alter the pulmonary microenvironment and trigger pulmonary inflammatory responses and subsequent fibrous scarring [3]. Overall, the prognosis of PF is poor. Furthermore, no drug is available that can reverse PF nor improve the deteriorated pulmonary function. PF resolution remains a substantial clinical challenge.

The COVID-19 pandemic in 2020 caused millions of deaths worldwide. This high mortality rate is known to be associated with acute lung injury, PF and further acute respiratory distress syndrome (ARDS); yet, there is currently no effective drug treatment for these pathological conditions. Data from previous coronavirus infections such as SARS and MERS, as well as current clinical evidence from COVID-19, support that SARS-CoV-2 infection may lead to pulmonary fibrosis, which seriously affects patient prognosis and quality of life [4].

Recently, cell-based therapies, especially stem cell therapies, have evolved into a promising therapeutic field. Mesenchymal stem cells have a variety of characteristics: (a) self-proliferating and differentiating potential; (b) abundant resources, such as bone marrow, adipose tissue and placenta; (c) low immunogenicity and in vitro transplantation; (d) chemotaxis and homing function; (e) immune regulation and tissue repair; (f) having, to date, elicited no serious adverse reactions in clinical trials of allotransplantation [5, 6]. These characteristics are critical for MSCs as a treatment for PF.

Among various animal models of pulmonary fibrosis (bleomycin, FITC, silica, radiation, etc.), bleomycin model is the most widely used animal model with better characteristics at present. The advantages of BLM-induced pulmonary fibrosis are easy induction, short induction period and good reproducibility However, a key shortcoming of the bleomycin model is the self-limiting nature of fibrosis, which contrasts with the typical progressive chronic fibrosis in the known natural history of human IPF [7]. Despite its limitations, the many significant advantages of the model and its record of illuminating in vivo mechanisms and the usefulness of drug discovery make the bleomycin model a valuable asset for preclinical and mechanistic research. At present, there are many preclinical studies on the treatment of PF by MSCs, but few or incomplete reviews have summarized them. In addition, some published reviews on the mechanism of MSCs in the treatment of PF lack relatively complete experimental evidence, or focus on some aspects of the mechanism of MSCs. With the globalization of COVID-19, the consequences of PF will increase the economic burden, so there is an urgent need for new and effective treatments. Although there have been no preclinical studies of MSCs in the treatment of virus-induced lung injury and PF, there are many clinical trials of MSCs in patients with COVID-19. Therefore, the purpose of this study was to systematically review preclinical studies, explore the effectiveness of MSCs in the treatment of BLM-induced experimental pulmonary fibrosis and analyze the potential mechanism, combined with clinical trials of current MSCs for COVID-19 and IPF, so as to provide support for clinical research and clinical transformation of MSCs.

## Materials and methods

### Search strategy

We performed electronic searches for topically-relevant studies from two literature databases: PubMed (− 2021.4) and Embase (− 2021.4). Search terms included (“exp Stem cells OR exp Bone Marrow Cells OR exp Stem Cell Transplantation OR exp Bone Marrow Transplantation AND exp Lung Diseases, Interstitial OR exp Pulmonary Fibrosis OR 'lung, bleomycin':ab”) or (“mesenchymal stem cells, pulmonary fibrosis, bleomycin”), without language restriction.

### Inclusion criteria

(a) The model of PF was induced by BLM; (b) Mice or rats were used as experimental animals, without immune deficiency or genetic modification; (c) BLM was delivered via intratracheal infusion; (d) The intervention was MSCs therapy, excluding embryonic stem cells and induced pluripotent stem cells; (e) The transplantation routes of MSCs included intratracheal infusion or intravenous injection; (f) The study reported on original data; (g) The primary end points were pulmonary histopathological changes and quantitative or semi-quantitative analysis of the degree of pulmonary inflammation or fibrosis.

### Data extraction

For the included studies, the following information were extracted: experimental animal type, sample size, BLM dose, source of MSCs, transplantation dose, transplantation time and route, and outcome observation time. The result data were extracted from the results text sections of these articles, as well as the accompanying figures and tables.

## Results

**Fig. 1:**
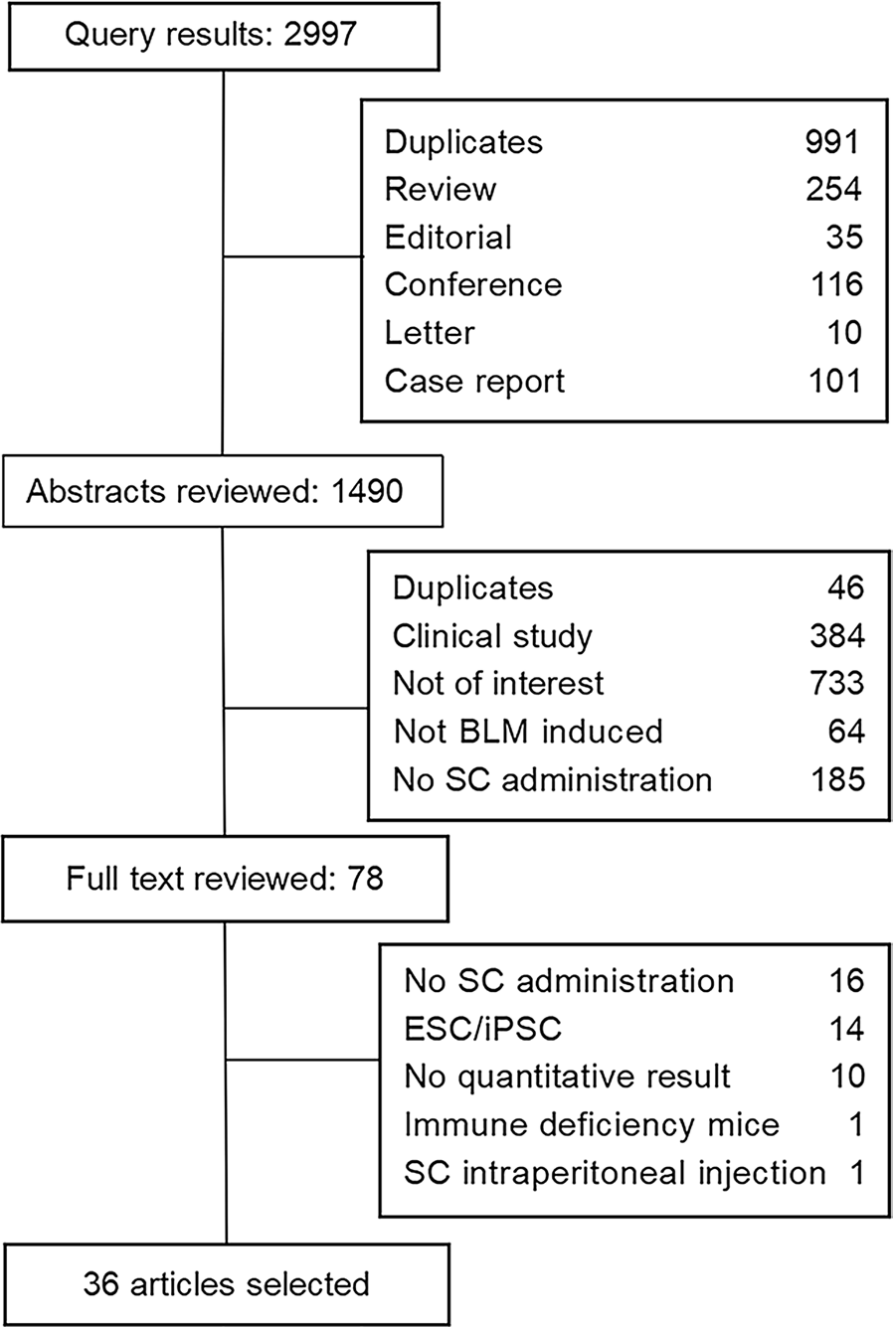
Flow chart of the study screening and selection for inclusion in review. BLM, bleomycin; ESC, embryonic stem cell; iPSC, induced pluripotent stem cell; SC, stem cell

A total of 2997 articles were identified with potential topical relevance and retrieved, of which 78 were identified as not being repeat publications, reviews, editorials, conference abstracts, case reports, clinical studies nor other irrelevant literature types. Finally, 36 studies were determined to meet all inclusion criteria, and were selected for analyzing in this review (Fig. 1) [8–43].

### Characteristics of the studies

All 36 studies had used animal models of mice (C57BL/6 in 23 studies; Swiss-Albino in 1 study) or rats (Sprague–Dawley in 8 studies; Wistar Albino in 3 studies; Fisher in 1 study). Most of the studies had used one-time intratracheal BLM infusion to induce PF, and only one study established the PF model by repeated intratracheal BLM infusions [36]. The MSCs used in the studies were derived from a variety organs from humans and rodent. The most common were MSCs derived from human bone marrow or umbilical cord tissues. Transplantation of the MSCs had been achieved by intratracheal or intravenous infusion. The MSCs transplantation times ranged from 0 to 21 days after BLM induction (immediate transplantation < 1 day in 13 studies; 1 to 7 days in 21 studies; > 7 days in 8 studies). Among them, 7 studies compared the effects of MSCs transplantation at different time periods on BLM-induced pulmonary inflammation and fibrosis (Table 1). The end point times of the studies ranged from 7 to 49 days after BLM induction. The end point indicators included pathological changes in lung tissue, content of inflammatory cells, inflammation-associated cytokines, fibrosis-associated proteins or cytokines in the lung, and Ashcroft score (Table 2).

**Table 1:** Characteristics of eligible studies of MSCs for BLM-induced pulmonary fibrosis

Author, year [References]	Number of samples	animal	Cell source	BLM dose	Number of injected cells (× 10^6^)	Transplant time after modeling (route)	Outcome time
Jiang, 2015 [8]	15	12–16 g C57BL/6 mice	AMSCs	2.5 mg/kg	1	1 h (IV)	7 d
Moradi, 2017 [9]	7	6–8 w C57BL/6 mice	hUMSCs	2 mg/kg	0.5	15 min (IT)	21 d
Orlando, 2019 [10]	8	12–16 w C57BL/6 mice	hUMSCs	1.5 mg/kg	0.25	24 h and 7 d (IV)	8,14,21 d
Llontop, 2017 [11]	6–8	300–360 g SD rats	AMSCs	2.5 mg/kg	2	2 or 14 d (IT)	28 d
Shi, 2018 [12]	4–5	6–8w C57BL/6 mice	mice DASCs	3 mg/kg	1	7 d (IT)	14,21 d
Reddy, 2016 [13]	10	10–12 w Swiss-albino mice	human AMSCs	4 mg/kg	1.8–2.2	3 and 6 and 9 d (IV)	24 d
Tashiro, 2015 [14]	8	22 m C57BL/6 mice	C57BL/6 mice AMSCs	2.5 mg/kg	0.5	1 d (IV)	21 d
Rathinasabapathy, 2016 [15]	8	8 w SD rats	SD rat AMSCs	2.5 mg	1	3 or 7 d (IV)	14 d
Cargnoni, 2020 [16]	26	8–9 w C57BL/6 mice	hAM-MSCs	2.3 mg/kg	1	15 min (IT)	2,4,7,9,14 d
Chu, 2020[17]	12	8 w SD rats	hUMSCs	5 mg/rat	25	21 d (IT)	49 d
Cahill, 2016 [18]	5	6–8 w C57BL/6 mice	Murine BMSCs	2 mg/kg	1	6–8 h or 9 d (IV)	28 d
Chen, 2020[19]	5	6–8 w C57BL/6 mice	MenSCs	3 mg/kg	0.5	2 and 7 d (IV)	21 d
Lan, 2015 [20]	6	8 w C57BL/6 mice	Murine BMSCs	1.5 mg/kg	0.5	3 d (IT)	7,21 d
Moroncini, 2018 [21]	8	12–16 w C57BL/6 mice	hUMSCs	1.5 mg/kg	0.25	24 h and 7 d (IV)	8,14,21 d
Huleihel, 2017 [22]	6	8–10 w C57BL/6 mice	BMSCs	2.15 mg/kg	0.5	7 d (IV)	14 d
Lan, 2017 [23]	≥ 5	8 w C57BL/6 mice	Murine BMSCs	1.5 mg/kg	0.2	3 d (IT)	7, 21 d
Zhao FeiYan, 2019 [24]	3–5	C57BL/6 mice	Murine BMSCs	3.5 mg/kg	0.1, 0.3, 1	14 d (IV)	21 d
Chu, 2019 [25]	12–20	SD rats	hUMSCs	8 mg/kg	5, 25	21d (IT)	49 d
Gad, 2020 [26]	10	8 w Wistar albino rats	Rat BMSCs	5 mg/kg	1	14 d (IV)	42 d
Yu, 2015 [27]	6	C57BL/6 mice	Murine BMSCs	5 mg/kg	2.5	1 or 3 or 6 d (IV)	28 d
Aguilar, 2009[28]	–	8 w C57BL/6 mice	C57BL/6 male MSCs	2 mg/kg	0.5	8 h and 3 d (IV)	14 d
Garcia, 2013 [29]	6–8	8–12 w C57BL/6 mice	hAFSCs	1.5 mg/kg	1	2 h or 14 d (IV)	3, 14, 28 d
Gazdhar, 2013 [30]	10	240–280 g Fisher F344 rats	Fisher F344 BMSCs	1.28 mg/rat	3	7 d (IT)	14, 21 d
Huang, 2014 [31]	4	200–240 g Wistar rats	Wistar rat BMSCs	5 mg/kg	2.5	0 or 7 d (IV)	7,14,28 d
Jun, 2012 [32]	5–7	C57BL/6 mice	C57BL/6 mice luMSCs	–	0.15–0.25	0 d (IV)	14 d
Kumamoto, 2009 [33]	5	6–8 w C57BL/6 mice	C57BL/6 BMSCs	3 mg/kg	0.5	3 d (IV)	10 d
Lee, 2010 [34]	6	6 w SD rats	Rats BMSCs	3 mg/kg	1	4 d (IV)	7, 14, 21, 28 d
Min, 2015 [35]	5	C57BL/6 mice	hUMSCs	40 mg/kg	–	0 d (IV)	7,14,28 d
Moodley, 2013 [36]	8	8 w C57BL/6 mice	hBMSCs, hAM-MSCs	0.15 mg(0,7 days)	1	10 d (IV)	17,31 d
Ono, 2015 [37]	6	8 w C57BL/6 mice	hBMSC	1 mg/kg	0.5	1 d (IV)	14 d
Ortiz, 2003 [38]	7	6–10 w C57BL/6 mice	Mice BMSCs	–	0.5	0 or 7 d (IV)	14 d
Zhao, 2008 [39]	5	200–250 g SD rats	Rat BMSCs	5 mg/kg	5	12 h (IV)	14 d
Cui, 2007 [40]	8	6w SD rats	Rat BMSCs	5 mg/kg	2.5	1 or 7 d (IV)	28 d
Li, 2013 [41]	20	6–8 w C57BL/6 mice	hUMSCs	2.5 mg/kg	1	3 d	21 d
Huang, 2012 [42]	6	8w Wistar rats	Rat BMSCs	5 mg/kg	2.5	0 d (IV)	7,14,28 d
Wang, 2013 [43]	5	180–200 g SD rats	hUMSCs	5 mg/kg	1	0 d (IV)	7,14,28 d

*DASCs* distal airway stem cells, *AMSCs* adipose-derived mesenchymal stem cells, *hAM-MSCs* human amniotic membrane mesenchymal stem cells, *hBMSCs* human bone marrow-derived mesenchymal stem cells, *hUMSCs* human umbilical cord mesenchymal stem cells, *AFSCs* amniotic fluid stem cells, *MenSCs* menstrual blood derived mesenchymal stem cells, *luMSCs* resident lung mesenchymal stem cells, *IT* intratracheal, *IV* intravenous, *g* gram, *m* month, *w* week, *d* day, *h* hour, *min* minute, – no statistics

**Table 2:** Summary of quantitative research results in MSCs for BLM-induced pulmonary fibrosis

Author, year [References]	Lung collagen (Hydroxyproline/ Collagen1α1/ Soluble collagen)	Ashcroft score	Mortality	Weight	BALF, total cell count	Lung IL-1	Lung IL-6	Lung TNF-α	Lung αSMA	Lung TGF-β	Lung MMP-2	Lung MMP-9	Lung MMP-13	TIMP	CTGF
Jiang, 2015 [8]	↓(< 0.05)^a^					↓(< 0.05)		↓(< 0.05)			↓^e^	↓^e^			
Moradi, 2017 [9]	↓mRNA (< 0.05)^b^			NS					NS	↓(< 0.05)					
Orlando, 2019 [10]	↓(< 0.05)^a^ ↓mRNA(< 0.05)^b^			↑^e^											
Llontop, 2017 [11]		↓(< 0.05)				NS	↓(< 0.05)			NS					
Shi, 2018 [12]	↓(< 0.01)^a^	↓(< 0.01)	↓(< 0.01)	NS					↓(< 0.01)						
Reddy, 2016 [13]	↓(< 0.001)^a^	↓(< 0.01)^d^	↓(< 0.001)			↓^e^		↓^e^		↓^e^	↓^e^		↓^e^	↓^e^	↓^e^
Tashiro, 2015 [14]	↓(< 0.05)^a^	↓(< 0.01)						↓mRNA < 0.05)		↓(< 0.05)	↓(< 0.01)				
Rathinasabapathy, 2016 [15]	↓(< 0.05)^a^	↓(< 0.05)					↓(< 0.05)							↓(< 0.05)	↓(< 0.05)
Cargnoni, 2020 [16]	↓mRNA(< 0.05)^b^					↓(< 0.05)			↓mRNA (< 0.01)						
Chu, 2020[17]	↓mRNA(< 0.05)^b^			↑(< 0.05)	↓(< 0.05)				↓(< 0.05)			↑(< 0.05)			
Cahill, 2016[18]		↓(< 0.001)				↓mRNA(< 0.05)									
Chen, 2020[19]	↓(< 0.01)^a^ ↓(< 0.05)^b^	↓(< 0.001)		↑(< 0.001)	↓(< 0.001)	↓(< 0.01)	↓(< 0.01)		↓(< 0.05)	↓(< 0.001)					
Lan, 2015 [20]	NS^c^	↓(< 0.05)				NS	NS								NS
Moroncini, 2018 [21]	↓(< 0.01)^a^, ↓(P < 0.001)^b^	↓(< 0.05)				NS	↓ < 0.001)		↓mRNA(< 0.01)	↓(< 0.01)					
Huleihel, 2017 [22]	NS^b^	NS	↓^e^	NS											
Lan, 2017 [23]	↓(< 0.05)^c^	↓(< 0.05)			↓(< 0.05)	NS	NS			↓mRNA(< 0.05)		↓mRNA(< 0.001)		↓(< 0.05)	↓mRNA(< 0.01)
Zhao, 2019 [24]	↓(< 0.01)^a^														
Chu, 2019 [25]	↓(< 0.05)^a^			↑(< 0.05)	↓(< 0.05)				↓(< 0.05)		NS	↑mRNA(< 0.05)			
Gad, 2020 [26]							↓(< 0.05)	↓(< 0.05)	↓(< 0.05)	↓(< 0.05)					
Yu, 2015 [27]										↓(< 0.05)		↓(< 0.05)		↓(< 0.05)	
Aguilar, 2009 [28]	NS^a^ ↓mRNA(< 0.05)^b^	NS^d^													
Garcia, 2013 [29]	↓ (< 0.05)^a^	↓ (< 0.05)			↑^e^									NS	
Gazdhar, 2013 [30]	↓ (< 0.05)^a^	↓ (< 0.01)													
Huang, 2014 [31]	↓ (< 0.05)^a^														
Jun, 2012 [32]		↓ (< 0.05)	↓^e^	↑ (< 0.05)											
Kumamoto, 2009 [33]	↓ (< 0.05)^c^	↓ (< 0.01)	↓(< 0.05)	↑^e^	↓ (< 0.05)										
Lee, 2010 [34]	↓ (< 0.05)^c^		↓(< 0.05)	↑^e^	↓ (< 0.05)	↓ (< 0.05)	↓ (< 0.05)	↓ (< 0.05)		↓(P < 0.05)					
Min, 2015 [35]	↓ (< 0.05)^a^ ↓mRNA(< 0.05)^b^	NS				NS	NS	NS		NS	↓ (< 0.05)	↓ (< 0.05)		↓ (< 0.05)	
Moodley, 2013 [36]	↓ (< 0.05)^a^					↓(< 0.05)	↓(< 0.001)	↓(< 0.05)		↓(< 0.05)	NS	↑(< 0.001)	NS		
Ono, 2015 [37]	↓ (< 0.01)^c^									↓ (< 0.01)					
Ortiz, 2003 [38]	↓ (< 0.05)^a^										mRNA↓^e^		mRNA↓^e^		
Zhao, 2008 [39]	↓ (< 0.01)^a^									↓ (< 0.01)					
Cui, 2007 [40]	↓ (< 0.05)^a^														
Li, 2013 [41]		↓ (< 0.05)									↓(< 0.05)			↑(< 0.05)	
Huang, 2012 [42]	↓ (< 0.01)^a^	↓ (< 0.01)													
Wang, 2013 [43]	↓ (< 0.05)^a^	↓ (< 0.05)								↓ (< 0.05)					

Numbers in parentheses indicate *p* values. ↓ or ↑ indicate significantly decreased or increased protein levels and/or mRNA levels in BLM + MSCs group compared with BLM group. a: Hydroxyproline; b: Collagen1α1; c: Soluble collagen; d: modified Ashcroft’s score; e: No statistics; NS: not significant

*BALF* bronchoalveolar lavage fluid, *TNF-α* tumor necrosis factor alpha, *TGF-β* transforming growth factor beta, *αSMA* alpha smooth muscle actin, *MMP* matrix metalloproteinases, *TIMP* tissue inhibitor of metalloproteinase, *CTGF* connective tissue growth factor

### Histopathology

A total of 31 studies performed hematoxylin–eosin (HE) staining of the lung tissues. Among these, 30 of the studies showed that, compared with normal lung tissue, BLM induction was followed by alveolar wall thickening, alveolar cavities occlusion, alveolar architecture destruction, and inflammatory cell infiltration in the lung interstitium or around the airway and blood vessels, including that of macrophages, neutrophils and lymphocytes. However, the MSCs transplantation led to significantly reduced inflammatory cell infiltration, alveolar occlusion and alveolar septal thickening [8–12, 15, 17–21, 23–27, 30–43]. The remaining study detected no significant improvement in lung tissue inflammation (by HE staining) in the MSCs treatment group compared with the BLM group [22].

Moroncini et al. [21] continuously evaluated the pathological changes of lung tissue in the BLM group and the MSCs treatment group. On day 8 after BLM induction, HE staining showed that inflammatory cells had infiltrated around the airway and blood vessels, and that the alveolar septum had become thickened; On day 14, the infiltration of inflammatory cells in the alveoli and interstitium increased progressively, accompanied by progressive destruction of alveolar structures and formation of fibrous bundles. On day 21, the infiltration of inflammatory cells decreased, the alveolar structure was obviously destroyed, and a large number of fibrous bundles were deposited in the interstitium. Semi-quantitative analysis results showed that on days 8, 14 and 21, lung tissue inflammation scores in the MSCs treatment group were significantly lower than those in the corresponding BLM group (*p* < 0.05).

### Inflammatory cells or inflammation-related cytokines in the lung

Levels of inflammation-related cytokines in lung tissue or blood reflect inflammation. Seven studies had evaluated the number of inflammatory cells (namely neutrophils and macrophages) in alveolar lavage fluid, and six of them had found that the number of inflammatory cells was significantly higher in the BLM group than in the control group, being significantly reduced after the MSCs treatment (all *p* < 0.05) [17, 19, 23, 25, 33, 34], only one study found no significant difference [29]. Meanwhile, the expression of inflammatory factors in the lung (namely IL-1β, IL-6, and TNF-α) showed the similar trend. One study had evaluated the levels of inflammatory cytokines in blood of control group, BLM group and MSCs treatment group, and the results showed that the MSCs treatment could significantly reduce the BLM-induced inflammatory response [19]. These results suggest a potential immunomodulatory role for MSCs in alleviating pulmonary inflammation.

### Pulmonary collagen content and Ashcroft score

The degree of PF was assessed by measuring the content or gene expression of fibrosis-related proteins in lung tissue, including hydroxyproline (HYP), collagen 1α1, and soluble collagen. Thirty studies had evaluated the expression of fibrosis-associated proteins in lung tissue, and twenty-eight of them found that these proteins were significantly increased in the BLM group compared with the control group, being significantly reduced after the MSCs treatment (all *p* < 0.05) [8–10, 12–17, 19, 21, 23–25, 28–31, 33–40, 42, 43]. Although there were no significant difference in two studies [20, 22].

Collagen was labeled by Masson staining or Sirius red staining, and the degree of PF was further analyzed based on Ashcroft scoring, which was determined in twenty studies. Aschcroft scoring was a continuous numerical scale for determining the degree of fibrosis in lung specimens. Grading was scored on a scale from 0 to 8, using the average of microscope field scores [44]. Seventeen of them showed that the Ashcroft scoring were significantly lower in the MSCs treatment group than that in the BLM group (all *p* < 0.05) [11–15, 18–21, 23, 29, 30, 32, 33, 41–43]. Three studies found no significant difference in the Ashcroft score between the MSCs and the BLM groups [22, 28, 35]. Besides, three studies analyzed the level of PF by measuring the staining area of pulmonary fibers, and all showed that the area of PF in the MSCs treatment group was significantly reduced compared with that of the BLM group (*p* < 0.05) [17, 25, 26]. Moroncini et al. [21] had found that on days 8, 14 and 21 after BLM induction, the degree of PF was progressively increased based on the Ashcroft score and HYP content measurement. However, on days 8, 14 and 21, the Ashcroft score and HYP content of the MSCs treatment group were significantly lower than those in the corresponding BLM group (*p* < 0.05). Most of the studies showed that the degree of PF increased progressively after BLM induction, while treatment with MSCs could significantly inhibit the progression of pulmonary fibrosis.

### Cytokines associated with fibrosis

The pro-fibrosis cytokines, such as alpha-smooth muscle actin (α-SMA), transforming growth factor-beta (TGF-β), connective tissue growth factor (CTGF) and tissue inhibitor of metalloproteinase (TIMP), as well as anti-fibrosis cytokines, such as MMPs in lung tissues, were measured to reflect the pro-fibrosis and anti-fibrosis microenvironment in the lung. Eight studies had measured the α-SMA in lung tissue, and seven had a lower α-SMA in the MSCs group than that in the BLM group (all *p* < 0.05), indicating that the activity of myofibroblasts was significantly reduced after MSCs treatment [12, 16, 17, 19, 21, 25, 26]. TGF-β is a major fibrogenic cytokine that induces the conversion of fibroblasts to myofibroblasts, leading to the deposition of large amounts of collagen. Fifteen studies had evaluated the TGF-β in lung tissue, and twelve had a decreased expression of TGF-β in the MSCs group compared with the BLM group (all *p* < 0.05) [9, 14, 19, 21, 23, 26, 27, 34, 36, 37, 39, 43]. Three of 4 studies measured CTGF in lung tissue showed a significant reduction of CTGF after the MSCs treatment [13, 15, 23].

The MMPs are a group of zinc ion-dependent endopeptidases, featuring common biochemical properties and highly homologous structure. Their main role is to degrade ECM. However, imbalance between MMPs and TIMPs leads to abnormal degradation of ECM and promotes the process of PF. Eleven studies had measured the levels of MMP-2, MMP-9, or MMP-13 in lung tissue, eight presented a decrease in MMPs levels in the MSCs treatment group compared with the BLM group [8, 13, 14, 23, 27, 35, 38, 41]. On the contrary, another three studies had shown an increased MMP-9 after the MSCs treatment (all *p* < 0.05) [17, 25, 36]. Five of seven studies measured TIMP in the lungs showed a significant decrease in TIMP expression after the MSCs treatment [13, 15, 23, 27, 35].

### Animal survival rate

Six studies had assessed the effect of MSCs therapy on survival, all showed improved survival followed MSCs therapy [12, 13, 22, 32–34]. Weight change of experimental animals had been evaluated in ten studies, and all had found it to have decreased significantly after BLM induction, However, seven of them had found no significant weight loss in the MSCs treatment group, which suggested MSCs treatment protected again weight loss after BLM treatment (all *p* < 0.05) [10, 17, 19, 25, 32–34].

### Timing of MSCs administration

Eight studies had compared the effect of MSCs transplantation on lung inflammation or fibrosis according to administrations at different time periods. Three studies had compared the effects of early transplantation (0–3 days) and delayed transplantation (7–14 days) on lung injury. Both the early and delayed treatment groups showed significant reductions in lung inflammation, collagen deposition, and Ashcroft score. Although lung function in the delayed treatment group was not complete restored [11, 15, 29]. Four studies found that the early treatment group (day 0 or 1) had significantly reduced PF score and collagen content, while the delayed treatment group (day 7 or 9) had no significant difference in the degree of PF compared with the BLM group [18, 31, 38, 40]. One study had performed MSCs transplantation on days 1, 3 and 6 after BLM induction, respectively, and the results showed that, compared with the day 1 treatment group, the day 3 and day 6 treatment groups had significantly inhibited BLM-induced pulmonary inflammation and PF, indicating that the delayed treatment with MSCs yielded a better therapeutic effect [27]. A possible reason was theorized as the lung injury having been more serious on the 3rd and 6th days, and the transplanted MSCs to have then migrated to the lungs to a greater extent under the action of chemokines, so as to exert a more profound therapeutic effect. Currently, there is no clear evidence to show a significant difference between early or delayed transplantation of MSCs in the treatment of BLM-induced lung inflammatory and fibrosis in rodents.

### Effects of MSCs on experimental acute lung injury or fibrosis in other species

We also retrieved some studies about the treatment of experimental lung injury or PF by MSCs in other species beyond rodents. One study found that human MSCs could reduce the severity of acute lung injury in a sheep model of bacterial pneumonia, human MSCs were well tolerated and improved oxygenation and decreased pulmonary oedema in a sheep model of severe ARDS [45]. Kong et al. [46] found that hUMSCs administration signifcantly improved therapeutic effects of linezolid on pneumonia resulted from MRSA infection in a rabbit model. In ex vivo perfused human lung injured by E.coli endotoxin, MSCs restored alveolar fluid clearance, reduced inflammation, and exerted antimicrobial activity, in part through keratinocyte growth factor secretion, to alleviate lung injury [47]. These results suggest that MSCs still have therapeutic effects on acute lung injury in species other than rodents.

## Discussion

Currently, no drugs have been approved for the treatment of fibrotic ILD, with the condition-specific exceptions of nintedanib and pirfenidone for the treatment of idiopathic PF. Immunoregulatory drugs such as glucocorticoids, immunosuppressants, and antioxidants reduce early lung damage and fibrosis by targeting growth factors and cytokines.However, these treatments can only slow the deterioration of lung function, and cannot reverse the process of fibrosis with many toxic side effects. Currently, lung transplantation is the only effective treatment strategy, but due to its complexity, multi-layered management and limited donor organ supply, PF remains a substantial challenge [48].

Due to the limited treatment options available, animal models of PF are still important tools to further study the pathogenic mechanisms affecting the occurrence and progression of the disease and to develop new anti-fibrosis strategies. Ortiz et al. [38] first reported the ability of systemically transplanted MSCs to improve BLM-induced PF in mice. Srour et al. [49] later reported a retrospective analysis of 17 studies (from 1996 to 2015.4) on MSCs-based treatment of BLM-induced PF in mice, showing that MSCs can improve the extent of BLM-induced pulmonary collagen deposition and reduce the Ashcroft score, decrease the total cell count, neutrophils count and TGF-β level in bronchoalveolar lavage fluid, and improve the survival rate. The method of our study is partially referenced Srour et al. A meta-analysis of six studies that investigated the effects of different administration times of bone marrow-derived MSCs on the treatment of BLM-induced PF in rat models showed that early (day 0) transplantation could prevent or reduce BLM-induced alveolar inflammation and PF, while late (day 14) transplantation could reduce alveolar inflammation. But, there was no clear evidence of improvement in PF [50]. Our study systematically analyzed published preclinical studies based on MSCs in the treatment of BLM-induced lung injury and fibrosis. Most of the findings indicated that MSCs treatment can reduce BLM-induced inflammatory response in lung tissue, reduce inflammatory cell infiltration and inflammatory cytokine expression, reduce ECM production and collagen deposition, and improve Ashcroft score. Therefore, a large number of preclinical studies have proved that MSCs have great therapeutic effect on acute lung injury and PF.

At the same time, clinical trials of MSCs in the treatment of PF are progressing (Table 3). We retrieved the current published clinical trials of MSCs for IPF, and preliminary findings indicate that, MSCs intravenous injection is feasible and safe for the treatment of patients with moderate to severe IPF. Most of the research shows that, at 6–15 months of follow-up, FVC, DLCO, 6 min walking distance (6MWD) and CT fibrosis score did not significantly change from baseline, and there was no evidence of worsening pulmonary fibrosis [51–54]. However, a clinical study of 10 patients with IPF showed that high-dose allogenic mesenchymal stem cell therapy is a safe and promising approach to reduce disease progression in patients with IPF and rapidly declining lung function [55]. Based on the systematic review of preclinical studies, combined with valid clinical data, MSCs can be used as a potentially promising means of treatment for IPF.

**Table 3:** Published clinical trials of MSCs in patients with IPF

Author [References]	Research time (clinical study staging)	Number of samples (the degree of IPF)	MSCs type	Number of injected cells (× 10^6^)	Evaluation index			Serious adverse reaction (follow-up time)
					Lung function(FVC, DLCO, PaO_2_)	6MWD	CT chest	
Chambers DC.[50]	2014 (phase Ib)	8 (moderate IPF)	Placenta-derived MSCs	1 or 2 /kg	No deterioration compared to baseline			No (6 months)
Tzouvelekis A.[51]	2010.6–2011.9 (phase Ib)	14 (mild to moderate IPF)	Adipose-derived MSCs	0.5 /kg (three times)	No deterioration compared to baseline			No (12 months)
Glassberg MK. [52]	2013.11–2014.10 (phase I)	9 (mild to moderate IPF)	Bone marrow–derived MSCs	20 or 100 or 200	No deterioration compared to baseline			No (15 months)
Campo A. [53]	2013–2016 (phase I)	13 (mild to moderate IPF)	Bone marrow autologous MSCs	10 or 50 or 100	No deterioration compared to baseline			No (12 months)
Averyanov A. [54]	2019 (phase I)	10 (moderate to severe IPF)	Bone marrow MSCs	1600	Significant improvement for the 6-MWD and lung function compared to baseline			No (12 months)

*DLCO* diffusing capacity for carbon monoxide, *FVC* forced vital capacity, *6MWD* 6-min walk distance, *CT* computed tomography

It is important to consider that viral pneumonia, such as in SARS, MERS-CoV, H7N9 influenza, as well as SARS-CoV-2 infection, which associated with acute lung injury, PF and further ARDS, may cause long-term lung damage, especially fibrotic ILD that persists after the viral clearance. Although the majority of ARDS patients survive, a proportion of survivors develop fibroproliferative reactions characterized by the accumulation of fibroblasts and deposition of collagen and other ECM components in the lungs [56]. A 15-year follow-up study of 71 SARS patients showed that 4.6% had pulmonary interstitial abnormalities [57]. And one-third patients recovered MERS-CoV infection have detected PF on computed tomography during followed-up [58]. In addition, Chen et al. [59] reported 41 patients with H7N9 pulmonary infection for up to 1 year, and detected PF on computed tomography in 41.5% and parenchymal abnormalities in 51.2%, including ground glass or reticular changes. Epidemiological, viral immunological and current clinical evidence support that PF may also be a major complication in patients with COVID-19 [60]. A prospective cohort study followed up the discharged COVID-19 patients for 4 months, and imaging abnormalities were found in 108 of 171 patients (63%), mainly subtle ground-glass opacities; fibrotic lesions were observed in 33 of 171 patients (19%) [61]. Therefore, prevention and treatment of PF represent one of the main goals to reduce the severity of COVID-19, improve the prognosis, and reduce the risk and severity of PF when patients survive from COVID-19, and then to reduce the overall socio-economic burden.

Clinical trials of MSCs for the treatment of acute lung injury, severe and critically ill COVID-19 are ongoing and some progress has been made. A review retrieved clinical studies prior to 2020.8 on MSCs for the treatment of COVID-19 or ARDS. A total of 11 studies were included, clinical data showed MSCs can overcome the clinical challenges currently faced by SARS-CoV-2 infected patients, specifcally who are seriously ill and not responding to conventional therapies [62]. Phase I and II clinical studies have shown that MSCs transplantation is a safe and potentially effective method for the treatment of moderate to severe ARDS [63, 64]. Besides, recent exploratory clinical studies showed that MSCs treatment was associated with improvements in mortality, dyspnea and chest imaging findings for severe and critically ill COVID-19 patients [65–69]. And phase I and II clinical trials have also shown that intravenous MSCs infusion in patients with moderate and severe COVID-19 is safe and well tolerated [70, 71]. Thus, based on available clinical data, MSCs-based therapy may serve as a salvage and priority treatment option for treating severe and critical viral pneumonia SARS-Cov-2-infected patients; although, its mechanism remains to be further elucidated.

After in vivo transplantation, MSCs can homing to injured lung tissue, release a variety of paracrine factors and extracellular vesicles, regulate the function of immune cells, reduce local inflammatory response, inhibit fibrous proliferation and promote endogenous lung injury resistance. However, MSCs playing a role in resistance to PF through differentiation into epithelial cells remains controversial. Furthermore, MSCs treatment could significantly improve the indexes of oxidative damage such as malondialdehyde (MDA), superoxide dismutase (SOD), glutathione (GSH) and oxidized glutathione (GSSG) and had a protective effect on lung tissue [35].

Studies have shown that in the early inflammatory stage of lung injury induced by BLM, the expression of MMPs was enhanced and their activity was increased, while the expression of TIMPs was relatively decreased and the collagenolytic activity of lung tissue was increased. On the contrary, in the PF stage, the expression of MMPs was relatively decreased, while the expression of TIMPs was increased and the collagenolytic activity of lung tissue was decreased, suggesting that imbalance of MMPs/TIMPs affects the formation and development of PF [72]. In our review of the literatures, eight studies had measured the MMPs in lung tissue at 14–28 days after BLM induction, and showed that the secretion of MMPs was significantly increased after BLM treatment, while the MSCs treatment contribute to a significantly decrease of MMPs [8, 13, 14, 23, 27, 35, 38, 41]. Three other studies had measured the level of MMP-9, in particular, at 31–49 days after BLM induction, and showed that it was decreased in the BLM group, but was significantly increased in the MSCs treatment group [17, 25, 36]. In brief, in the early inflammatory stage of lung injury, MSCs can reduce the damage of MMPs to lung tissue by downregulating the expression of MMPs. In the late stage of fibrosis, MSCs up-regulate the expression of MMPs and inhibit the activity of TIMP, thus promoting the dissolution of MMPs on fibers. Therefore, MSCs can reduce lung injury and prevent the progression of fibrosis by regulating the content of MMPs and TIMP at different stages of lung injury.

**Fig. 2:**
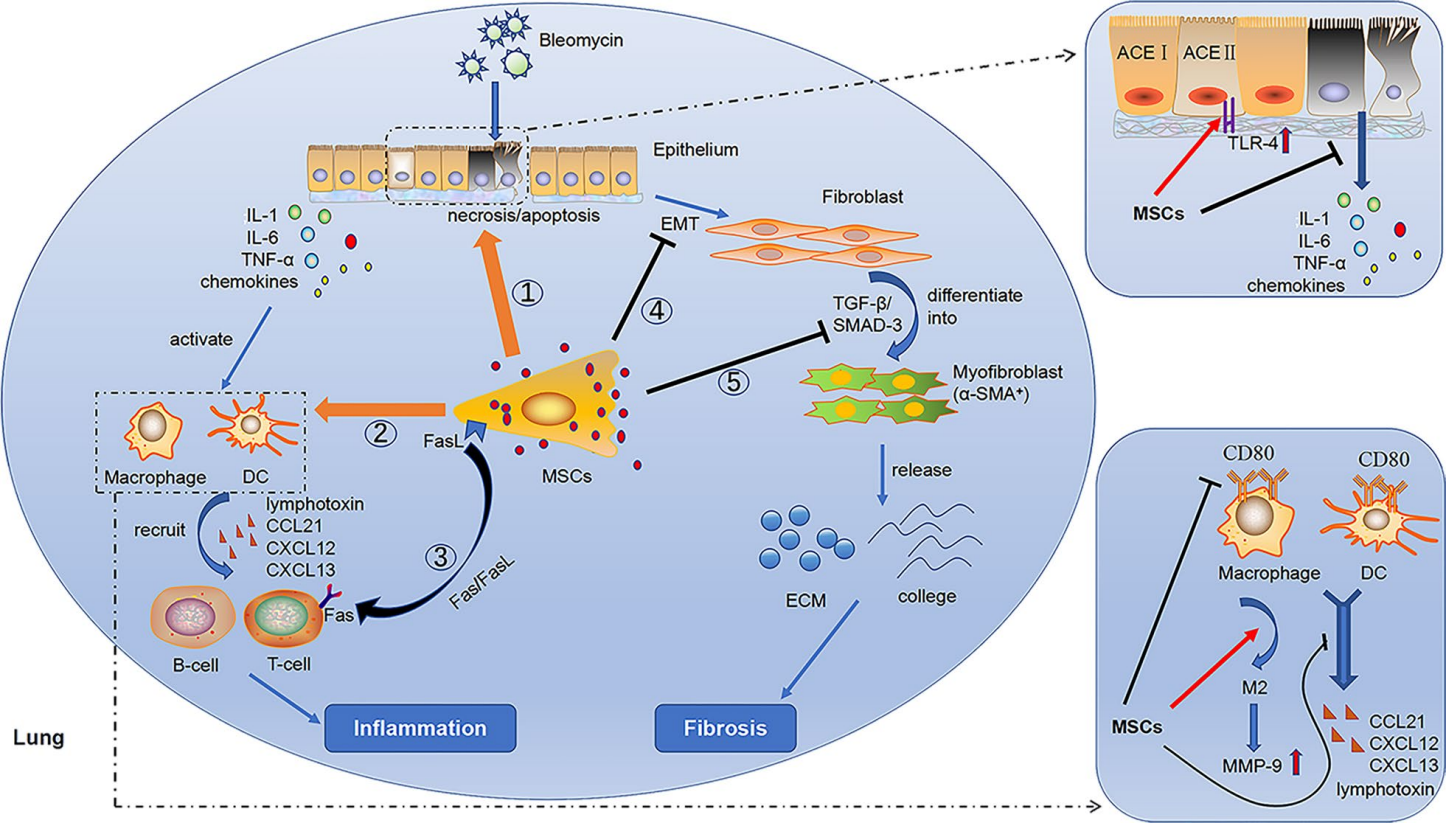
Potential mechanism of MSCs in the treatment of BLM-induced lung injury. ①MSCs reduce BLM-induced epithelial cell necrosis or apoptosis by inhibiting oxidative stress response, and further reduce the production of inflammatory mediators such as cytokines and chemokines. MSCs promote the expression of TLR-4 in alveolar epithelial cells, trigger the lung regeneration signal of HA-TLR-4, and promote the regeneration and repair of alveolar epithelial cells. ② Immunomodulatory effects of MSCs: MSCs reduce the expression and density of CD80 costimulatory molecules in macrophages and dendritic cells, and reduce their ability to induce antigen-specific T cell immune response; MSCs promote the transformation of macrophages to M2 phenotype, reduces the inflammatory potential, and increases the synthesis and secretion of MMP-9, which contributes to the degradation of extracellular matrix, including collagen; MSCs reduce macrophages and dendritic cells the expression of chemokine (lymphotoxin, CCL21, CXCL12, CXCL13), thus reducing the B cells to recruit; ③Direct induction of apoptosis of activated T cells through Fas/FasL signaling pathway alleviates abnormal excessive immune response; ④ MSCs inhibit epithelial mesenchymal transformation (EMT); ⑤ MSCs inhibit the activation of TGF-β/SMAD-3 signaling pathway, thus inhibiting the activation of myofibroblasts and further reducing the synthesis of collagen and other extracellular matrix (ECM)

Potential mechanism was summarized in in Fig. 2. Concisely, the mechanisms of MSCs treatment for acute lung injury and PF including: (1) homing to injured lung tissue, reduce epithelial cell necrosis or apoptosis by inhibiting oxidative stress response; (2) regulate the immune response by promote the transformation of macrophages to M2 phenotype and reduce the expression of chemokine from macrophages and dendritic cells, further reducing inflammatory cell infiltration and inflammation-related cytokines content in the lung; (3) induce the apoptosis of activated T cells through Fas/FasL signaling pathway; (4) inhibit epithelial mesenchymal transformation, and (5) inhibit the activation of myofibroblasts and then to reduce the synthesis of collagen and ECM [73, 74].

In conclusion, based on the above encouraging findings from preclinical studies and clinical trials, MSCs can significantly improve BLM-induced PF, suggesting that MSCs can serve as a potential treatment for PF, including IPF and viral-induced PF. Most of the literatures included in the review performed MSCs transplantation in the inflammatory phase or early fibrotic phase (0–14 days), which the PF is not yet fully established, and only 2 studies performed MSCs transplantation at 21 days after BLM induction [17, 25]. Our results suggested that MSCs can reduce lung inflammation and inhibit the progression of PF, further studies are needed to determine whether MSCs can reverse established pulmonary fibrosis and improve pulmonary function.

## Data Availability

Availability of data and materials will be available through contact corresponding authors upon publication of the manuscripts.

## Abbreviations

AFSCs: Amniotic fluid stem cells
ARDS: Acute respiratory distress syndrome
AMSCs: Adipose-derived mesenchymal stem cells
BLM: Bleomycin
CTGF: Connective tissue growth factor
COVID-19: Coronavirus disease 2019
DASCs: Distal airway stem cells
ESC: Embryonic stem cell
hAM-MSCs: Human amniotic membrane mesenchymal stem cells
hBMSCs: Human bone marrow-derived mesenchymal stem cells
hUMSCs: Human umbilical cord mesenchymal stem cells
HE: Hematoxylin–eosin
HYP: Hydroxyproline
iPSC: Induced pluripotent stem cell
ILDs: Interstitial lung diseases
luMSCs: Resident lung mesenchymal stem cells
MSCs: Mesenchymal stem cells
MMPs: Matrix metalloproteinases
MDA: Malondialdehyde
MenSCs: Menstrual blood derived mesenchymal stem cells
PF: Pulmonary fibrosis
SARS: Severe acute respiratory syndrome
SOD: Superoxide dismutase
SC: Stem cell
TGF-β: Transforming growth factor-beta
TIMP: Tissue inhibitor of metalloproteinase
α-SMA: Alpha-smooth muscle actin
DLCO: Diffusing capacity for carbon monoxide
FVC: Forced vital capacity
6MWD: 6-Min walk distance
CT: Computed tomography
